# Respiratory Microbiome of Endangered Southern Resident Killer Whales and Microbiota of Surrounding Sea Surface Microlayer in the Eastern North Pacific

**DOI:** 10.1038/s41598-017-00457-5

**Published:** 2017-03-24

**Authors:** Stephen A. Raverty, Linda D. Rhodes, Erin Zabek, Azad Eshghi, Caroline E. Cameron, M. Bradley Hanson, J. Pete Schroeder

**Affiliations:** 1 British Columbia Ministry of Agriculture, Animal Health Centre, Abbotsford, BC Canada; 2 Institute for the Oceans and Fisheries, University of British Columbia, Vancouver, BC Canada; 3 Northwest Fisheries Science Center, National Marine Fisheries Service, NOAA, Seattle, WA USA; 4 Department of Biochemistry and Microbiology, University of Victoria, Victoria, BC Canada; 5 National Marine Mammal Foundation, San Diego, CA USA; 6 Marine Mammal Research Associates, Sequim, WA USA; 7 University of Victoria - Genome BC Proteomics Centre, Victoria, BC Canada

## Abstract

In the Salish Sea, the endangered Southern Resident Killer Whale (SRKW) is a high trophic indicator of ecosystem health. Three major threats have been identified for this population: reduced prey availability, anthropogenic contaminants, and marine vessel disturbances. These perturbations can culminate in significant morbidity and mortality, usually associated with secondary infections that have a predilection to the respiratory system. To characterize the composition of the respiratory microbiota and identify recognized pathogens of SRKW, exhaled breath samples were collected between 2006–2009 and analyzed for bacteria, fungi and viruses using (1) culture-dependent, targeted PCR-based methodologies and (2) taxonomically broad, non-culture dependent PCR-based methodologies. Results were compared with sea surface microlayer (SML) samples to characterize the respective microbial constituents. An array of bacteria and fungi in breath and SML samples were identified, as well as microorganisms that exhibited resistance to multiple antimicrobial agents. The SML microbes and respiratory microbiota carry a pathogenic risk which we propose as an additional, fourth putative stressor (pathogens), which may adversely impact the endangered SRKW population.

## Introduction

**Figure 1:**
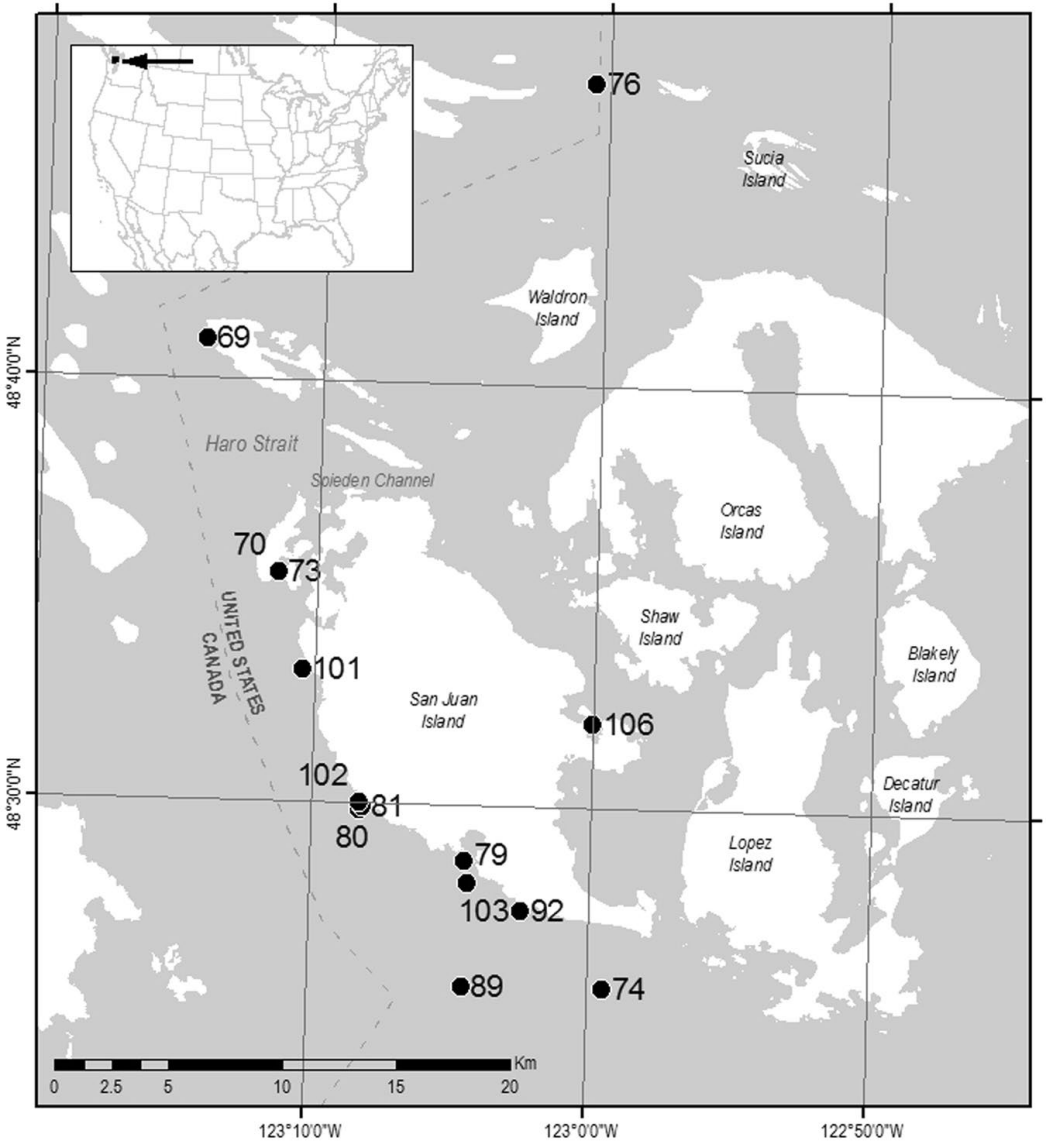
Map of study area displaying locations of waypoints for SML sampling, and the relationship of the study area to the continental US (inset). Map with waypoints were generated by B. Sylvander (NOAA Fisheries) using ESRI ArcGIS Desktop 10.3.1 (http://desktop.arcgis.com/en/arcmap/10.3/main/get-started/whats-new-in-arcgis-1031.htm).

Killer whales (*Orcinus orca*) are among the most widely distributed marine mammals in the world with higher densities in the highly productive coastal regions of higher latitudes. In the eastern North Pacific, the Southern Resident Killer Whale (SRKW) population ranges seasonally from Monterey Bay, California to the Queen Charlotte Islands, British Columbia. The movements of this endangered, demographically isolated population are largely associated with migratory salmon populations, frequenting the southern extent of the Salish Sea in the northeastern Pacific from spring through fall^1^. Since the initial census in 1974, the population increased from 70 to 98 individuals in 1995^2^. Over the last two decades, the population size has generally shown a negative trajectory (based upon counting individual known animals), punctuated by periods of abrupt decline and recovery^3^. As of December 2016, annual population census surveys estimate 78 SRKWs. Although killer whales are globally designated as “lower risk: conservation dependent” by the International Union for Conservation of Nature, the SRKW population has been listed as “endangered” under the Canadian Species at Risk Act (SARA) since June 2003 and under the United States (U.S.) Endangered Species Act (ESA) since November 2005. Within the Salish Sea, Washington State and inner waters of British Columbia (see Fig. 1), the SRKW population encounters urbanized waterways and a plethora of environmental stressors caused by humans. During the ESA listing and review process, three factors were identified as principal threats: reduced prey availability, high levels of anthropogenic contaminants, and disturbance by marine vessels and sound (Committee on the Status of Endangered Wildlife in Canada Assessment, August 28, 2009; U.S. Federal Register 70 FR 69903, November 18, 2005).

In 2011, the U.S. 5-year status review of SRKWs acknowledged that infectious diseases could be a high impact factor for recovery, but conceded that insufficient information was available to determine a direct impact^4^. Based on available microbiology isolates and necropsy findings of stranded sympatric porpoises and dolphins, infectious diseases are a principal clinical presentation and probable cause of death in beach-cast animals^3, 5, 6^. Recommendations to improve the overall health monitoring of the SRKW included development of a standardized necropsy protocol^7^ and the development of a Recovery Plan for SRKW^8^. However, the ability to characterize the health status of live SRKW is restricted by public sentiment, law and logistics, relying on opportunistic and non-invasive sample collection. The latter can be achieved by sampling the exhaled breath as an alternate means for acquiring samples indicative of the respiratory health of free-swimming whales.

Respiratory adaptations in cetaceans have evolved to consist of prolonged breath holding during deep dives followed by short, rapid expirations and inhalations with a tidal volume of 70–85 percent, and exhalation of 0.3 seconds for smaller cetaceans and 1–2 seconds for larger whales^9^. These respiratory patterns, coupled with a direct conduit from the blowhole to lungs, may facilitate inadvertent aspiration of small quantities of the sea surface microlayer (SML). The SML or neuston is the uppermost hydrophobic 1 mm of the ocean that is the interface between the atmosphere and water^10, 11^. Due to water movements, intimate contact with atmospheric oxygen, warmer temperatures and a variety of ocean-dwelling microbes, the SML is enriched in dissolved organic matter, lipids and microbiota, making it biologically and chemically distinct to the underlying pelagic seawater^12^. During porpoising, aerosolized SML laden with microorganisms and contaminants may be aspirated deep into the tracheobronchial tree and deposited into alveolar spaces with more forceful exhalation and inhalation respirations. Impaired host immune responses related to contaminant loads, reproductive status and other stressors may also contribute to proliferation and deeper tissue invasion of commensal bacteria resulting in respiratory disease. An assessment of the composition of microorganisms within both exhaled breath samples and the SML could reveal a potential relationship between host respiratory and SML microbial flora.

Research on lung microbiota has disproved the long-held belief of lungs as sterile organs^13^. A survey of lung microbiota in humans has revealed predominantly *Proteobacteria*, *Firmicutes*, and *Bacteroidetes* at the phylum level^13^ but little is known about the lung microbiota in killer whales. This study aimed to determine the microbial composition of the SML and exhaled breath samples of the SRKW population, to compare the microbial profiles of exhaled breath samples and SML of proximal waters, and to assess differences in microbial composition. Bacterial isolates from the SML and exhaled breath samples were also screened for direct evidence of antibiotic resistance, an important indicator of human activity and waste seepage into the marine environment. To our knowledge this is the first study to directly assess the respiratory and environmental pathogen burden within the SRKW population, and the study has the additional unique attribute of assessing this burden over a multi-year period.

## Results

### Animal Signalment

A total of 26 exhaled breath samples were collected and analyzed in this study. These samples included twelve identified SRKWs, five samples from individuals who could not be unequivocally identified, and two samples of mixed exhaled breath from unidentified individuals (Table 1). Among the twelve identified SRKWs, four animals were sampled more than once but in different years (Table 1). The mixed exhaled breath samples consisted of breath from a female and an adolescent (unidentified breath sample #11) and breath from two females (unidentified breath sample #12). Birth years ranged from 1951 to 1995, and at the time of sampling included eight males, seven females, and six animals of undetermined sex. Exhaled breath samples were collected from animals in the Salish Sea around the San Juan Islands of Washington State (Fig. 1). No adverse behavior or clinical disease was apparent before, during or after sampling. At the time of exhaled breath and SML sampling, representative air, human breath, and rain control samples were also collected.

**Table 1:** List of bacteria and fungi detected in exhaled breath samples collected from SRKW.

SRKW identifier	Sex	Birth Year	Bacteria	Fungi	Year sampled
J1	M	1951	*Vibrio alginolyticus*	—	2007
L7	F	1961	*Bacillaceae* sp.	*Penicillium brevicompactum*	2006
			*Rothia dentocariosa*		
			*Staphylococcus epidermidis*		
			*Staphylococcus pasteuri*		
			*Staphylococcus sp.*		
			*Staphylococcus xylosus*		
J14	F	1974	*Staphylococcus aureus*	—	2009
L41	M	1977	*Pseudomonas fluorescens*	*Aureobasidium pullulans*	2006
			*Staphylococcus cohnii cohnii*	*Penicillium sp.*	
L41	M	1977	*Burkholderia* sp.	*Cladosporium cladosporioides*	2007
			*Staphylococcus cohnii*		
L53	F	1977	*Arthrobacter* sp.	*Aureobasidium pullulans*	2007
			*Kocuria* sp.	*Phoma* sp	
			Mollicutes (PCR)		
K16	F	1985	*Arthrobacter* sp.	*Aureobasidium pullulans*	2008
			*Pseudomonas* sp.	*Penicillium expansum*	
			*Vibrio* sp.		
L74	M	1986	*Salmonella enterica* serovar Heidelberg	*Cladosporium cladosporioides Cladosporium* sp.	2007
			*Streptomyces* sp.		
L79	M	1986	*Halomonas marina*	—	2006
			*Halomonas sp.*		
			*Staphylococcus warneri*		
L79	M	1989	*Psychrobacter phenylpyruvicus*	*Alternaria* sp.	2007
L79	M	1989	*Bacillus barbaricus*	*Alternaria* sp.	2009
			*Brevibacterium* sp.	*Cladosporium* sp.	
				*Pleospora herbarum*	
L84	M	1990	*Bacillus simplex*	*Cladosporium cladosporioides*	2007
			*Curtobacterium pusillum*		
			*Microbacterium* sp.		
L85	M	1991	—	*Alternaria sp.*	2007
				*Cladosporium* sp.	
L87	M	1992	*Alteromonas sp.*	—	2007
			*Arthrobacter* sp.		
			*Bacillus sp.*		
			*Bacillus licheniformis Halomonas* sp.		
			*Rheinheimer* sp.		
			*Vibrio splendidus*		
L87	M	1992	*Bacillus* sp	*Aspergillus sp.*	2008
			*Pseudomonas* sp.	*Cladosporium*	
			*Stenotrophomonas* sp.	*cladosporioides*	
			*Vibrio wodanis*	*Penicillium chrysogenum*	
J30	M	1995	—	—	2007
J30	M	1995	—	*Coniochaeta lignaria*	2009
U (unidentified) 2	—	—	—	—	2006
U (unidentified) 3	—	—	—	—	2006
U (unidentified) 6	—	—	—	*Aureobasidium pullulans*	2006
U (unidentified) 7	—	—	—	—	2006
U (unidentified) 8	—	—	*Sporosarcina gensengisoli*	—	2008
			*Stentrohomonas sp.*		
U (unidentified) 11	F + adolescent	—	—	*Ascomycota sp.*	2009
				*Phoma sp.*	
U (unidentified) 12	F + F	—	Mollicutes (PCR)	*Phoma* sp.	2009
			*Psychromonas arctica*		
			*Vibrio pectenicida*		
			*Vibrio splendidus*		

### Microbiota of SRKW Exhaled Breath

A wide diversity of microbes was recovered from the killer whale exhaled breath and SML samples (Table 1 and Supplementary Table S1). Microorganisms were identified by a combination of culture characteristics such as morphology and Gram-stain, general PCR on microbial cultures or direct PCR on SML and exhaled breath samples. All listed microbes were absent from the corresponding environmental and laboratory control samples (Supplementary Table S2), indicating specificity for exhaled breath or SML source, rather than contamination during sample collection or processing. The most abundant bacteria belonged to the Staphylococcaceae family (eight detections in five animals, *Staphylococcus* sp.), Bacillaceae family (six detections in four animals, predominantly *Bacillus* sp.), and Vibrionaceae family (six detections in four animals, *Vibrio* sp.) and the most abundant fungi were the Pleosporaceae family (seven detections in five animals, predominantly *Alternaria* and *Phoma* sp.) and Davidiellaceae family (seven detections in six animals, *Cladosporium* sp.). Exhaled breath samples from two animals were positive for Mollicutes (L53 and U12) by PCR. In addition, no cytopathic effect (CPE) was observed in cell lines after three weeks incubation and PCR did not detect specified viral pathogens in exhaled breath samples or SML.

Bacteria recovered from exhaled breath samples and SML differed among animals and across sampling years for the same animal (Table 1). Based on the identified microbial species and associated virulence factors, many isolates were considered commensals or transient colonizers with minimal pathogenicity (Table 1). However, pathogenic bacteria were also detected within SRKW exhaled breath samples including *Staphylococcus epidermidis, S. aureus*, *Pseudomonas fluorescens*, and *Salmonella enterica* Heildelberg. These microbes have been implicated in human diseases, detected in stranded sympatric marine mammals, isolated in captive killer whale studies, and recovered from lesions identified during prior killer whale necropsies (Table 2)^14–16^.

**Table 2:**
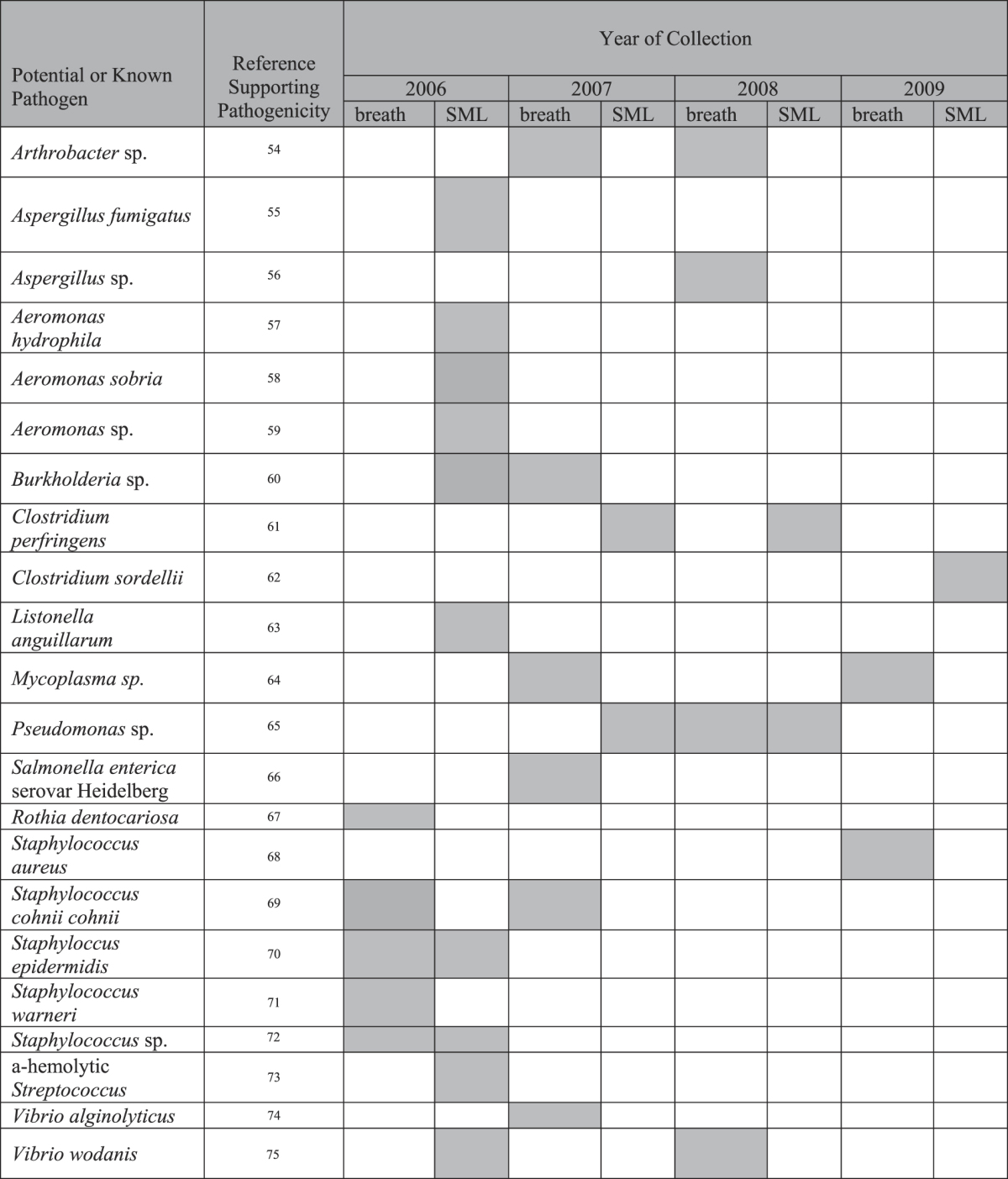
Potential and known human or animal pathogens identified in SRKW exhaled breath and SML samples^54–75^.

In contrast to the diversity of bacterial species detected in the exhaled breath samples, fungi cultured from 13 of 25 exhaled breath samples (52%) comprised a single phylum, *Ascomycota* (Table 1); *Cladosporium* sp. were detected in six samples (24%). No relationship was found between the identified fungi and animals, sampling year, sex or age. Additional fungal genera included pathogenic species, such as *Penicillium* spp, *P brevicompactum*, *P. chrysogenum, Phoma* spp, *Alternaria* spp, *Aspergillus* spp, and *Cladosporium cladosporioides* (Table 1 and Supplementary Table S1).

### Microbiota of Sea Surface Microlayer

During each sampling year, SML samples were collected during two different sampling periods and microbial culture of the SML yielded a preponderance of bacteria, with the highest detections for *Pseudoalteromonas* spp. (18 detections), *Vibrio* spp. (14 detections), and *Halomonas* spp. (11 detections). These three genera were identified in each sampling year and at the majority of waypoints (Supplementary Table S1). Both recognized pathogens, including coliforms indicative of fecal contamination, were identified via these analyses (Table 2) and environmental and commensal microbes such as *C. perfringens* and *Bacillus cereus* were identified in seawater samples (Supplementary Table S1).

SML fungal isolates were dominated by four genera, constituting 67% of the detections: *Cladosporium* spp. (19 detections), *Alternaria* spp. (12 detections), *Botrytis* spp. (11 detections), and *Epicoccum* spp. (11 detections).

### Microbial Community Structure

**Figure 2:**
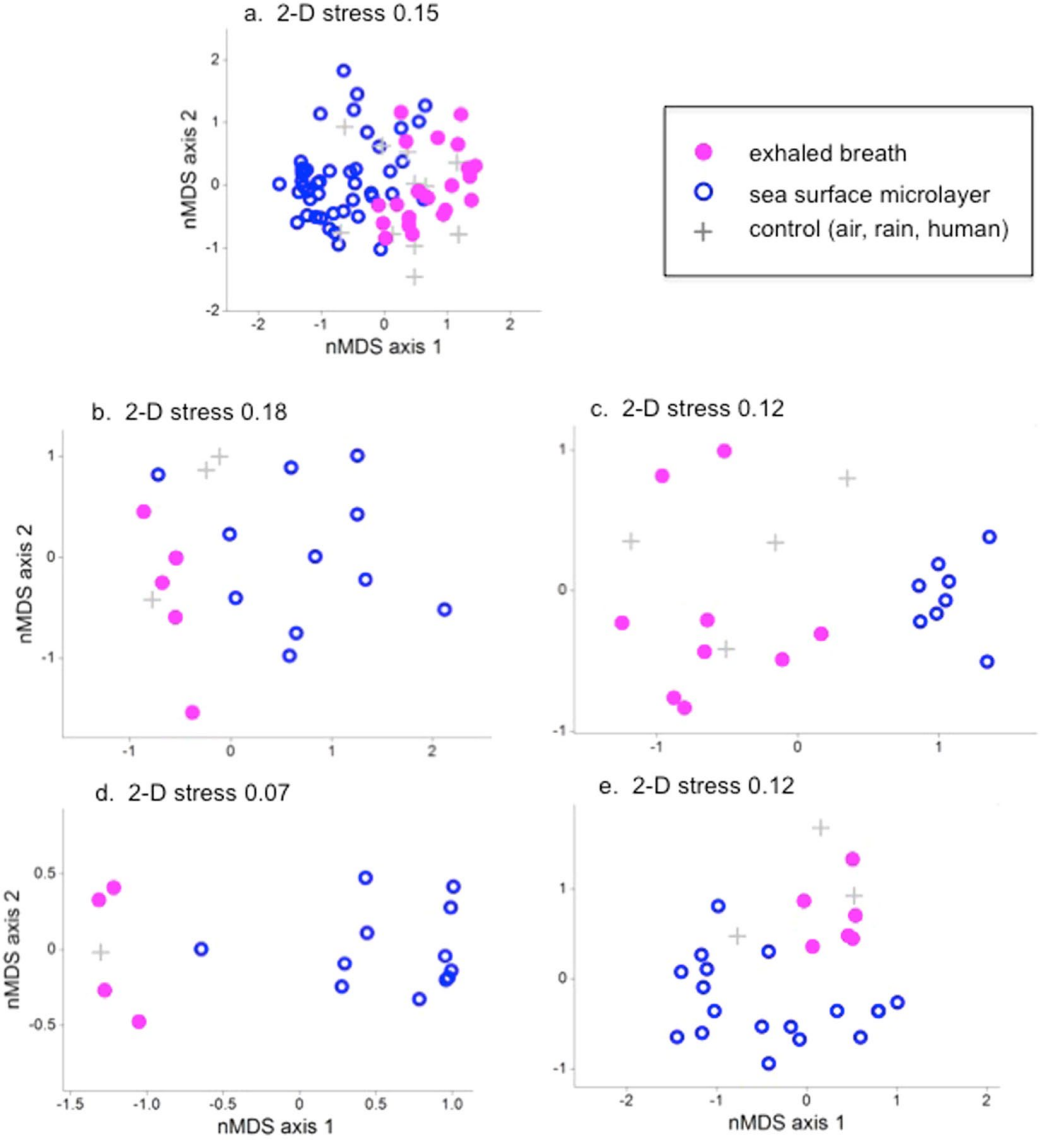
Non-metric multidimensional scaling plots of microbial communities identified in SRKW breath (pink closed circles), sea surface microlayer (blue open circles), and control samples (gray plus signs) for all four years (**a**), 2006 (**b**), 2007 (**c**), 2008 (**d**), and 2009 (**e**).

Fifty-six microbial genera were cultured from exhaled breath and SML throughout the sampling period. Phenotypic and genotypic analysis of exhaled breath- and SML-recovered microbes identified 35 species of bacteria comprising seven groups (*Flavobacteriacae*, *Micrococcoineae*, *Streptomycineae*, *Clostridium*, *Bacilli, Gammaproteobacteria, Betaproteobacteria*) and 17 fungal isolates from three taxa (*Basidiomycota*, Fungi *incertae sedis* and *Ascomycota*) (Supplementary Fig. S1). For the breath samples, there was no significant effect of gender on bacterial community structure (t = 0.478, p_perm_ = 0.698). Age of the animal also did not have an effect on community structure (t < 1.2005, p_perm_ > 0.127 for all pair-wise comparisons). Although there appeared to be a difference between years for breath communities, the corresponding control samples also differed (Supplementary Table S5), indicating that apparent differences between years may have been due to differences in ambient microbe sampling. Non-metric multidimensional scaling, similarity analysis, and permutational multivariate analysis of variance of microbial community structures revealed several observations. Although breath, SML, and control samples displayed some community overlap (Fig. 2), breath samples were distinct from SML samples both in community dispersion and centroid distances (Supplementary Table S4), suggesting that exhaled breath samples were not simply aerosolized SML or sea water. SML and control communities were consistently distinct from each other (Supplementary Table S4), suggesting minimal sampling or processing contamination for these samples. However, exhaled breath and control communities were less distinct, indicating some risk of environmental contamination during sample collection.

### Detection of Antibiotic Resistant Bacteria

**Figure 3:**
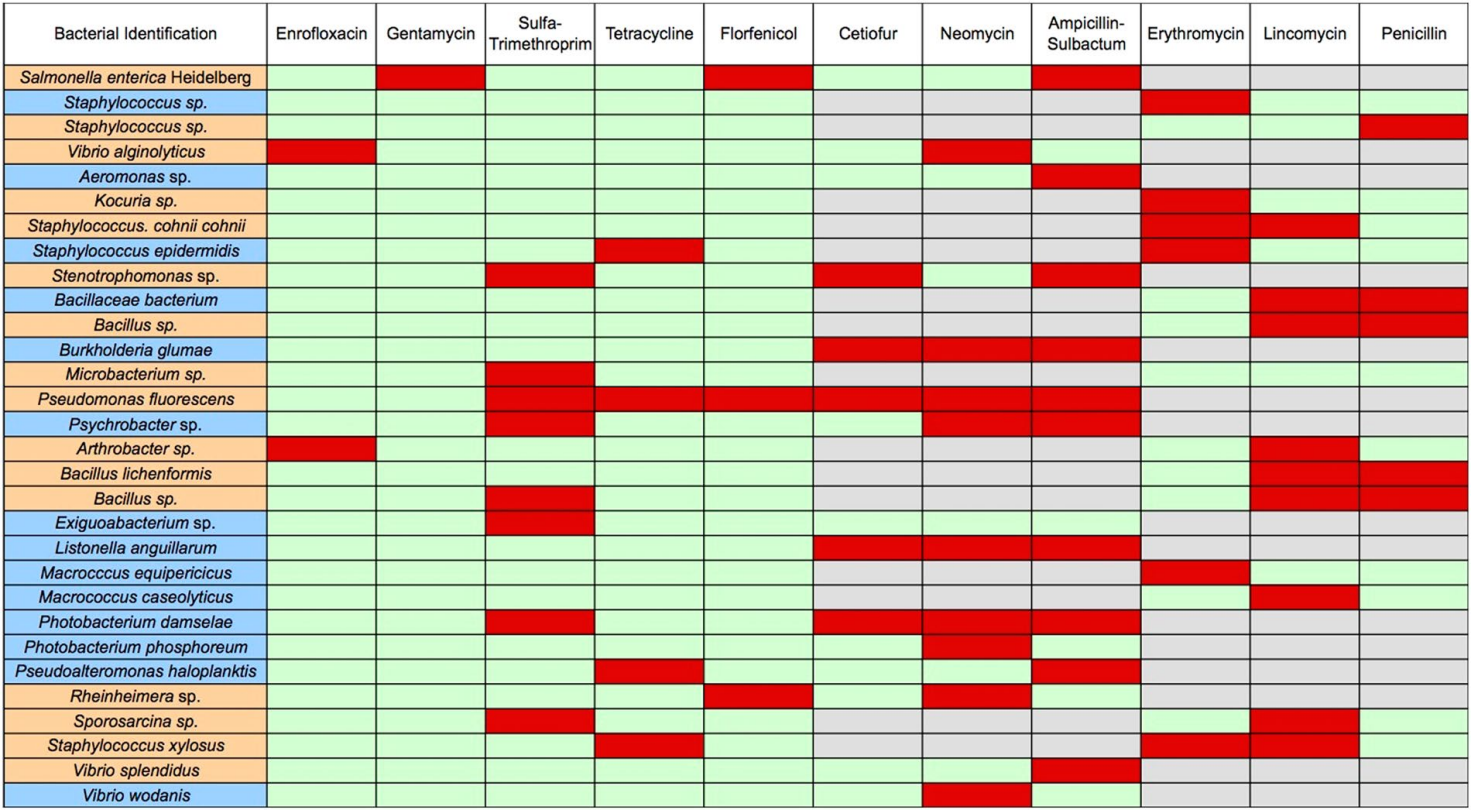
Heat map of antibiotic resistance detected in breath and SML bacteria. Bacterial names in orange boxes are from exhaled breath samples, and names in blue boxes are from SML samples. Matrix cells in red signify resistance, matrix cells in green signify sensitivity, and matrix cells in gray indicate no testing because the antibiotic is not appropriate for the bacterium.

Antibiotic susceptibility testing revealed multiple antibiotic resistant Gram-positive and Gram-negative bacteria from both the SRKW exhaled breath plumes and the SML (Fig. 3). Gram-positive bacteria from SRKW exhaled breath plumes and from SML featured increased resistance to erythromycin, lincomycin and penicillin with some isolates resistant to sulfamethoxazole-trimethroprim and tetracycline. In general, screened bacteria were susceptible to florfenicol, gentamycin and enrofloxacin (Fig. 3). Similarly, SRKW and SML samples contained Gram-negative bacteria that displayed general susceptibility to gentamycin and enrofloxacin, pronounced resistance to neomycin and ampicillin-sulbactum, moderate resistance to cetiofur, sulfamethoxazole-trimethroprim and florfenicol, and minimal resistance to tetracycline (Fig. 3). Resistance patterns of recovered bacteria varied by year and between SML and exhaled breath samples.

## Discussion

This study is the first to characterize the microbial contents of exhaled breath from a wild population of killer whales. SRKW are subjected to stressors of declining prey availability, anthropogenic contaminants, and human-caused acoustic disturbances. The observations reported herein examined the respiratory microbiome for both commensal and potentially pathogenic microorganisms in this endangered species. Furthermore, the study identified antibiotic resistant bacteria in both seawater samples and killer whale exhaled breath samples, a finding that suggests human waste contamination of the marine environment. Using a novel approach to assess the potential for environmental pathogen exposure, the study establishes key baseline information regarding microbial flora of the killer whale upper respiratory system via: (1) sampling wild, individual free-swimming SRKWs at a population level; (2) annual sampling of the population over 4 years to identify exhaled breath and SML microbial variation in space and time; (3) repeated individual sampling to determine if the microbial composition evolves over time; and (4) comparison of the microbial profile in the SML with that of SRKW exhaled breath samples to assess the extent of pathogen exposure and recruitment within the host.

Our methodology captured a single exhaled breath of a surfacing SRKW, in its critical habitat, a snapshot that presumably under-represents the actual microbial profile of the respiratory tract. Further, a portion of our SRKW exhaled breath and SML samples were analyzed using culture-dependent methodologies, which have the inherent limitation of only detecting readily culturable organisms; fastidious organisms, labile or unculturable organisms may not be identified using these analysis methods. In spite of these limitations, it is clear that we identified a significant and varied repertoire of microorganisms from both the SRKW exhaled breath and SML samples, in particular in the former samples. Exhaled breath and SML communities were different from each other, indicating that the breath samples were not merely aerosolized seawater. Control samples were included to account for potential contamination from the atmospheric environment in the vicinity of the surfacing whale, and culture controls were included to capture potential contamination from laboratory manipulation sources. The strong dissimilarity between SML and control samples indicates that cross-contamination in laboratory processing was unlikely. But the weaker distinction between exhaled breath and control samples suggest the possibility that some of the detected microorganisms originated during sampling, in spite of efforts to minimize inoculation from the ambient atmospheric environment.

Culture-dependent studies to establish baseline microbial flora and potential pathogen exposure have been undertaken in bottlenose dolphins and results have demonstrated a profound difference in bacterial and fungal burdens between more heavily impacted and less polluted environments^17, 18^. In our study, culture-dependent and culture-independent PCR-based analyses of SRKW exhaled breath and SML samples identified 35 bacterial genera and 17 fungal genera (Supplementary Fig. S1). Bacterial species of particular concern for marine mammals found in exhaled breath samples included *Pseudomonas aeruginosa* and *Staphylococcus aureus*, both of which have been implicated in pulmonary disease in marine and terrestrial mammals^19, 20^. Moreover, competitive exclusion is likely an important component of mucosal integrity and some isolates recovered from exhaled breath samples may be representative of this process. As pneumonia has been commonly diagnosed in killer whales at necropsy^21^, characterization of respiratory commensals and pathogens is critically important. Additional microbes of concern in SRKW exhaled breath samples included pathogenic *Vibrio alginolyticus* ^22^ and opportunistic pathogenic species of *Aspergillus spp.* ^23^, which are associated with mortality in *Rachycentron canadum* ^22^ and pulmonary disease in bottle nose dolphins^23^, respectively, and have previously been recovered from other stranded and live captured marine mammal species^24–26^.

Analysis of the SML microbiome revealed predominantly *Pseudalteromonas* spp and *Vibrio* spp with fewer, but more diverse, microbial genera in contrast to isolates recovered from SRKW exhaled breath samples. This is consistent with previous analyses of SML from the North Sea which also detected *Pseudoalteromonas* and *Vibrio* spp.^27^. Microbes of concern for marine mammals found in the SML included *Clostridium perfringens*, α-hemolytic *Streptococcus* sp., and *Aspergillus fumigatus*, which are pathogenic to humans and marine mammals^28–32^. In particular, *C. perfringens* was consistently detected in sampling years 2006, 2007, and 2008, indicating this pathogen is a significant and persistent component of the SML.

Because of the health threats posed by *Brucella* sp., *Mycoplasma* sp., *Cryptococcus gattii* and morbillivirus, which have previously been associated with or pose a theoretical risk to morbidity in stranded cetaceans in the region, molecular and culture studies were undertaken to screen for these specific agents. In our analyses we detected samples that were positive for Mollicutes and *Mycoplasma* sp., but we did not detect *Brucella* sp., *Crytococcus gattii* or morbillivirus^33, 34^.

Further analysis of the SRKW exhaled breath and SML sample isolates identified 17 of 26 Gram-negative and 18 of 27 Gram-positive bacteria that were resistant to multiple antimicrobial agents. Of particular interest, 7 of 26 Gram-negative and 3 of 27 Gram-positive bacteria were resistant to 3 or more human and veterinary antibiotics. These bacterial genera can exhibit extensive multidrug resistance and possess sophisticated mechanisms for acquiring and transmitting plasmid-mediated multidrug resistance to other bacterial species^33–37^. In our studies, the greatest degree of antimicrobial resistance was observed to both second line antibiotics (e.g. lincomycin) and antibiotics widely used in the fields of human medicine (e.g. neomycin, ampicillin-sulbactum) and veterinary companion and production animal medicine (cetiofur, penicillin, erythromycin).

The detection of bacterial strains within SRKW respiratory samples that are resistant to multiple antibiotics has several important implications. First, it may provide evidence for the seepage of land-based antimicrobial compounds and/or antibiotic resistant microorganisms into the marine environment and bioaccumulation, and possible biomagnification, into the highest marine trophic level. Second, the occurrence of several recognized species, such as *Pseudomonas* and *Staphylococcus*, that commonly exhibit resistance to multiple antimicrobial compounds is of concern, since these bacteria have a propensity to acquire and transmit antimicrobial resistance genes, and multiple antibiotic resistance can increase microbial virulence. Thus, multiple SML antimicrobial resistant microbes could potentially expand and spread resistant bacterial species within the marine environment and colonize exposed marine fauna, increasing the risk of disease in the critical habitat of the SRKW. Third, detection of multiple antibiotic resistant bacteria in this natural setting has significant medical implications for humans who may recreate and work within this marine environment and consume seafood potentially contaminated with multi-antibiotic resistant organisms.

With regards to the potential source of the multiple antibiotic resistant microorganisms, it is noteworthy that within 30 miles of the study area the city of Victoria, British Columbia does not have a secondary sewage treatment facility, and instead discharges primary treatment product from the resident population of approximately 360,000 to the Salish Sea. Studies have shown that sewage treatment plants remove 99% of the antibiotic resistant bacteria^35^ and 99.8% of antibiotic resistance genes from sewage^38^. A correlation may exist between the lack of secondary treatment for sewage entering the Salish Sea and the presence of antibiotic resistant bacteria within SRKW breath and SML samples. Support for this association comes from prior studies demonstrating that antibiotic resistant bacteria present in seawater isolates increase in frequency in areas of human habitation without secondary sewage treatment facilities^39^.

The endangered SRKW population is diminished relative to historic census information. Current numbers are 78 individuals, with negligible growth since monitoring of this population was initiated 40 years ago^40^. Seventy five free-ranging SRKW have died since the beginning of 1998, highlighting the vulnerable status of SRKW. The population faces multiple risks, including human-related activities, ecosystem alterations due to climate issues, pollution, contamination, urban development, overfishing and decreased availability of prey and its size, and exposure to infectious pathogens while experiencing the cumulative effects of multiple chronic stressors.

Individual and population health status reflects a complex and dynamic interplay of environment, agent and host factors. Host and environmental microbial flora can be potential stressors that are linked with the individual health and population health of SRKWs, and these findings should be placed in the context of ongoing threats confronted by killer whales regionally and worldwide. These threats include increased shipping traffic, noise, prey depletion, contaminants, endocrine disruptors and other factors. The combined and cumulative impact of these threats could cause decreased immune system function and thus further increase the susceptibility of these animals to environmental or host adapted microbes^41^. By linking these data with previous publications reporting histopathology and microbiology findings from necropsies of both free-ranging and captive killer whales and sympatric Odontocetes, it is possible to assess the potential threat of infectious organisms to the health of the free-ranging endangered SRKW. Exhaled breath sample collection via a fixed (or telescoping) pole, or through the use of remotely controlled unmanned aircraft systems (UAS or drones), may provide additional noninvasive opportunities to assess and monitor health through pathogen screening as well as hormone quantification, characterization of metabolite derivatives, photogrammetry and thermal imaging that can be conducted alongside and complementary to ongoing biopsy and fecal collections^42, 43^. Continued investigations into the role of pathogens and infectious diseases in the decline of the SRKW population will better define the magnitude of the problem and improve formulation of a comprehensive conservation plan to preserve this endangered population.

## Materials and Methods

### Sampling Locations

Breath and environmental sampling was conducted within an area bounded by latitudes 48°41′30″N and 48°17′00″N and longitudes 122°50′00″W and 123°15′00″W (Fig. 1). Collections were opportunistic and coincident with nearshore SRKW movements during the summer and fall over a four-year period: August 31–October 20, 2006; September 15–October 16, 2007; June 23–October 6, 2008; and September 10–September 18, 2009. Due to inclement weather and seasonal movements, the SRKWs are generally inaccessible during much of the late fall and winter. Geographic sample locations were predominantly within the Salish Sea, and sites adjacent to low urban or agricultural development were designated as control environments.

### Sample Collection, Processing, Bacterial Speciation and Culture Analyses

All activities conducted in proximity to SRKW were performed in accordance with approved animal handling protocols under NOAA and DFO permits and SARA Scientific license. A whale was approached from behind and to one side of the animal and the vessel was maneuvered to time the approach so that the petri plates attached to an aluminum pole were positioned approximately 0.4–0.6 m above the blowhole and into the exhaled plume when an orca surfaced to exhale. Between 20–50% of the approaches resulted in successful positioning to collect a sample; the success rate for collecting a breath sample during a successful approach was 100%. The identity of individual SRKWs have been catalogued for the last 40 years by the Center for Whale Research (http://www.whaleresearch.com) and breath sampled individuals were identified by expert personnel with reference to distinct markings, pigmentation patterns, nicks and healed scars and comparison with published catalogues^40^. A veterinary clinician (J. Pete Schroeder) with extensive marine mammal and cetacean medicine experience attended field sampling efforts to visually assess animals and collect breath and SML samples. Whales exhibiting signs of clinical disease or distress were not approached, nor were female-calf pairs approached. No adverse or aversion behavior was noted throughout the duration of the field work. Exhaled breath samples from SRKWs were collected directly onto petri dishes to facilitate microbial isolation, subsequent bacterial and fungal identification, antibiotic sensitivity testing, metagenomics, and molecular screening for recognized pathogens.

Petri plates with selective or non-selective agars were affixed to the 7.62 m long telescoping aluminum pole using suction cups (Supplementary Fig. S2 panel A). To minimize contamination by water droplets and ambient air, petri dish lids were taped at a single point to facilitate rapid opening and closing of the lids by rotating the pole. The pole with fastened petri dishes was passed through the exhaled breath plumes of surfacing SRKWs for exhaled breath sample collection (Supplementary Fig. S2 panel B). Three of the four or five attached petri dishes contained media, including Tryptone soy agar (TSA) supplemented with 2% NaCl, Columbia Blood agar and Sabouraud agar (SAB). The NaCl-supplemented media was used for recovery of halophilic microbes. After exposure to the exhaled breath, the pole was retracted and agar plates were removed, wrapped with parafilm and chilled for a maximum of 55 hours while *en route* to the laboratories for analysis. Upon receipt, the agar plates were incubated under varying temperatures and conditions. Columbia blood agar was incubated at 35 °C+/−2 °C in 5–10% CO_2_; TSA with 2% NaCl was incubated at 15 °C+/−2 °C and SAB agar at 30 °C+/−2 °C. Cultures were incubated for varying lengths of times and frequently observed for bacterial and fungal growth. The success rate for obtaining growth upon culturing was 100% if samples were introduced to media within the 55 hour timeframe from collection of exhalate to laboratory culturing.

Two empty petri dishes were also affixed to the telescoping pole to obtain exhaled breath droplets and aerosolized condensate for direct polymerase chain reaction (PCR; see below) analyses and for further microbiology and attempted virus isolation. SRKW exhaled breath samples collected on empty petri dishes were swabbed in the field with a sterile swab presoaked with sterile distilled water, then placed into either an empty sterile transport tube (for direct PCR analysis; Falcon tubes, Fisher Scientific, Pittsburgh, PA) or a sterile tube containing one of the following growth media; selenite broth, Luria Broth, Luria Broth supplemented with 3.5% NaCl, M9 minimal media or M9 minimal media supplemented with 3.5% NaCl. For selective enrichment for *Salmonella*, samples inoculated into selenite broth were incubated at 42 °C+/−2 °C for 24 hours, then transferred to XLT-4 agar and Hektoen agar and incubated at 35 °C+/−2 °C under aerobic conditions. Suspect *Salmonella* colonies were sub-cultured onto Columbia blood agar for biochemical and serological testing. The remaining samples inoculated into growth media were transported from the field to the lab and upon arrival, were re-incubated at ambient temperature with shaking until visible turbidity was observed. Cultures were then streaked on plates containing the same growth media used for liquid growth and plates were grown for an additional 72 hours at ambient temperature. When necessary, cultures were re-streaked to obtain single colonies. After each sampled whale breath, control air samples were collected at the same time as the SRKW exhaled breath samples by exposing a series of agar plates described above to the air, then processed in an identical manner to breath and SML samples.

To collect SML samples, sterile Plexiglas sheets were placed on the sea surface during calm sea conditions, and surface tension adhering water samples were transferred into sterile containers using a sterile squeegee and funnel (Supplementary Fig. S3). Whole water SML samples were shipped on wet ice and processed within 30 hr post-collection. On receipt at the laboratories, SML samples underwent bacterial and fungal analysis utilizing selective and non-selective microbiological methodologies. SML samples were also submitted to an ISO 17025 accredited laboratory (I.G. MicroMed Environmental Inc, Richmond, BC) for standard colony forming units (CFU) analysis of total and fecal coliforms, *Escherichia coli*, *Salmonella* sp., *Pseudomonas aeruginosa*, *Pseudomonas* spp., *Campylobacter* spp., *Vibrio* spp., and fungi including yeast and molds using their Standards Council of Canada-approved, proprietary methodology (http://www.igmicromed.com/micromed-water-testing-services.html). Aliquots from samples (SRKW exhaled breath and SML) were also inoculated into Madin Darby canine kidney (MDCK) and VERO cells using conventional techniques, incubated for 3 weeks and assessed for viral cytopathic effect (CPE).

### Laboratory Analyses

Direct PCR screening (without culturing) was performed on exhaled breath samples for morbillivirus^44^, canine distemper virus, influenza virus^45^, *Brucella* spp^46^. and *Mycoplasma* spp. (Mollicutes)^47, 48^. A similar direct PCR screening approach has been used previously to identify bacteria in animal and environmental samples^49^. Bacterial and fungal identification were performed by a variety of conventional laboratory methods determined by the collaborating facility or investigator performing the analysis. Bacteria or fungal isolates obtained from the TSA plates supplemented with 2% NaCl, Columbia blood agar, SAB agar and *Salmonella* selective agar were identified based on colony morphology, growth characteristics, gram stain and biochemical testing. In some instances, bacterial or fungal identification was performed using bacterial 16S rRNA and fungal 18S rRNA sequencing (broad PCR as opposed to direct PCR performed on samples without culturing microorganisms). Nucleic acids were extracted from isolates using the QiaAMP® DNA Mini kit following the manufacturer’s protocol (Qiagen Inc., Toronto, ON, Canada) and 2 µl of this template was used in a 25 µl PCR reaction. The 16S rRNA gene was amplified using the Uni-C and Uni-D primers (Supplementary Table S3) using the following conditions; 1 cycle at 95 °C for 4 min, 30 cycles of 95 °C for 1 min 45 sec, 57 °C for 90 sec, 72 °C for 2 min 15 sec; and 1 cycle at 72 °C for 7 min. The 18S rRNA gene was amplified utilizing external and internal fungal primers Fun-A/B and Fun 1/2 utilizing the following conditions; 1 cycle at 95 °C for 5 min, 50 cycles of 95 °C for 60 sec, 50 °C for 60 sec, 72 °C for 60 sec; and 1 cycle at 72 °C for 7 min. The amplified PCR product was then sequenced and compared to known sequences in Genbank. In the University of Victoria laboratory, individual colonies of bacterial and fungal isolates from streak plates were re-suspended in 25 µl H_2_O and 3 µl of the suspension was used as template in a 50 μl total reaction volume for colony PCR. The PCR reactions amplified bacterial 16S rRNA and fungal 18S rRNA genes using the following conditions; 1 cycle at 94 °C for 3 min, 30 cycles of 45 sec at 94 °C, 50 sec at 56 °C and 90 sec at 72 °C; and 1 cycle at 72 °C for 10 min. The primers used in the PCR reactions are listed in Supplementary Table S3. For identification of microorganisms, PCR amplicons were submitted for DNA sequencing. Sequences were then manually curated and aligned with either microbial or fungal genomes using Basic Local Alignment Search Tool (BLAST) against the National Center for Biotechnology Information (NCBI) database. Correct taxonomic assignment was confirmed by screening selected sequences against the GreenGenes database^50, 51^.

### Antibiotic Sensitivity Testing

Antibiotic sensitivity was determined for bacteria isolated from SRKW exhaled breath samples and SML water samples using the standard Kirby-Bauer disk diffusion assay^52^.

### Microbial Community Analysis

Counts for the lowest taxonomic level assignments from all diagnostic assays were used to build a Bray-Curtis similarity matrix. For PCR-based detections with binomial outcomes, a count of one or zero (presence or absence, respectively) was assigned. All counts were fourth root transformed, and a zero-adjusted Bray-Curtis dissimilarity matrix generated (Supplementary Table S6)^53^. The matrix was used for non-metric multidimensional scaling (nMDS), analysis of similarity (ANOSIM), and permutational multivariate analysis of variance (PERMANOVA) for each year of sampling, as well as for all four years combined, to test for the relatedness of exhaled breath samples to respective SML and control samples. Using subsets of the matrix, the effects of gender, age, and year of sampling were tested by PERMANOVA and ANOSIM. Due to sample size, ages were aggregated into categories (1 to 20, 21 to 30, and >30 years old). Tests for effects of year of sampling included control samples to account for potential sampling contamination. Analyses were performed initially using R software (v. 3.2.0; vegan package; R Foundation for Statistical Computing, Vienna, Austria) and subsequently with PRIMER (v. 7; multidimensional scaling, PERMDISP,ANOSIM, and PERMANOVA; PRIMER-E Ltd, Lutton, Ivybridge, UK).

## Electronic supplementary material

Supplementary Data File

Supplementary Table S6
