# Supplementary material for: Respiratory Microbiome of Endangered Southern Resident Killer Whales and Microbiota of Surrounding Sea Surface Microlayer in the Eastern North Pacific
Source: Sci Rep. 2017 Mar 24;7:394. doi: 10.1038/s41598-017-00457-5 (PMC5428453; doi:10.1038/s41598-017-00457-5)
Supplement: Supplementary file 1 — Supplementary Data File [file 41598_2017_457_MOESM1_ESM.pdf]

Title: Respiratory Microbiome of Endangered Southern Resident Killer Whales and Microbiota of Surrounding Sea Surface Microlayer in the Eastern North Pacific

Authors: Stephen A. Raverty[1,2*], Linda D. Rhodes[3], Erin Zabek[1], Azad Eshghi[4§], Caroline E. Cameron[4], M. Bradley Hanson[3], and J. Pete Schroeder[5,6]

[1]British Columbia Ministry of Agriculture, Animal Health Centre, Abbotsford, BC, Canada

[2]Institute for the Oceans and Fisheries, University of British Columbia, Vancouver, BC, Canada

[3]Northwest Fisheries Science Center, National Marine Fisheries Service, NOAA, Seattle, WA, USA

[4]Department of Biochemistry and Microbiology, University of Victoria, Victoria, BC, Canada

[5]National Marine Mammal Foundation, San Diego, CA USA

[6]Marine Mammal Research Associates, Sequim, WA, USA

[§]Current affiliate: University of Victoria - Genome BC Proteomics Centre, Victoria, BC, Canada

*To whom correspondence should be addressed: 1767 Angus Campbell Road,

Abbotsford BC, V3G 2M3; phone, 604 556-3003; fax, 604-556-3010; e-mail, Stephen.Raverty@gov.bc.ca

Supplementary Table S1. List of bacteria and fungi detected in sea surface microlayer samples from indicated waypoints. Microbes detected in the corresponding control samples were considered possible contaminants and were not included in the table.

Waypoint Identifier	Year	First Sampling		Second Sampling	
		Bacteria	Fungi	Bacteria	Fungi
69	2006	*Halomonas sp.*<br>*Pseudoalteromonas sp.*<br>*Staphylococcus epidermidis*		*Aeromonas sp.*<br>*Moritella marina*<br>*Moritella sp.*<br>*Photobacterium phosphoreum*<br>*Pseudoalteromonas haloplanktis*<br>*Pseudoalteromonas sp*<br>*Pseudomonas fluorescens*	*Aspergillus fumigatus*
69	2007	*Alteromonas sp.*	*Alternaria sp.*<br>*Botrytis sp.*<br>*Epicoccum sp.*<br>*Cladosporium sp.*<br>*Verticillium sp.*	*NS*	*NS*
69	2008	*Acinetobacter haemolyticus*<br>*Alteromonas sp*<br>*Halomonas sp.*<br>*marine proteobacterium*<br>*Marinomonas sp*<br>*Pseudoalteromonas sp*<br>*Psychrobacter sp*<br>*Vibrio sp.*	*Alternaria sp*<br>*Cladosporium sp*<br>*Mucor spp*<br>*Penicillium sp*	---	*Alternaria sp.*<br>*Aureobasidium sp.*<br>*Fusarium sp.*
69	2009	*Alteromonas sp.*<br>*Clostridium bifermentans*<br>*Clostridium sordellii*<br>*Pseudoalteromonas sp.*<br>*Pseudomonas fluorescens*	*Epicoccum sp.*<br>*Cladosporium sp.*	---	---
70	2006	*Halomonas marina*<br>*Halomonas sp.*<br>*Psychrobacter sp*<br>*Streptococcus alpha-hemolytic*	---	*Aeromonas hydrophila*<br>*Burkholderia glumae*<br>*Exiguobacterium sp*<br>*Halomonas sp.*<br>*Listonella anguillarum*<br>*Pseudoalteromonas haloplanktis*<br>*Pseudoalteromonas sp*<br>*Psychrobacter immobilis*<br>*Vibrio wodanis*<br>*Vibrio sp.*	*Penicillium namyslowski*
70	2007	*Clostridium perfringens*<br>*Clostridium sp.*<br>*marine proteobacterium*<br>*Pseudomonas fluorescens*<br>*Pseudomonas sp.*<br>*Vibrio sp.*	*Alternaria sp.*<br>*Botrytis sp.*<br>*Epicoccum sp.*<br>*Cladosporium sp.*	*NS*	*NS*
70	2008	*Cobetia sp.*<br>*Halomonas sp.*<br>*marine proteobacterium*<br>*Pseudoalteromonas sp.*<br>*Psychrobacter sp.*	*Aureobasidium sp.*<br>*Mucor sp.*<br>*Penicillium sp.*<br>*Trichoderma sp.*	*Alteromonas sp.*<br>*Clostridium perfringens*<br>*Cobetia sp.*<br>*Halomonas sp.*<br>*Pseudoalteromonas sp.*	*Aureobasidium sp.*<br>*Botrytis sp.*<br>*Epicoccum sp.*
70	2009	*Clostridium bifermentans*<br>*Clostridium sordellii*	*Epicoccum sp.*<br>*Cladosporium cladosporioides*<br>*Cladosporium sp.*	---	---
73	2006	*Aeromonas sobria*<br>*Escherchia coli*<br>*Staphylococcus epidermidis*<br>*Staphylococcus sp.*	---	NS	NS
74	2006	---	---	*Burkholderia glumae*<br>*Pseudoalteromonas sp*<br>*Pseudomonas fluorescens*	*Aspergillus fumigatus*
76	2006	---	---	*Pseudomonas fluorescens*	---
79	2006	---	---	*Burkholderia glumae*	---

				Photobacter damselase<br>*Pseudoalteromonas artica*<br>*Pseudoalteromonas sp*<br>*Pseudomonas stutzeri*<br>*Psychrobacter sp*<br>*Vibrio logei*<br>*Vibrio tasmaniensis*	
80	2007	---	---	NS	NS
81	2006	*Aeromonas sobria*<br>*Bacillaceae sp*<br>*Bacillus simplex*<br>*Enterobacter cloacae*<br>*Escherichia coli*<br>*Macrococcus caseolyticus*<br>*Macrococcus equipericicus*<br>*Moritella marina*<br>*Photobacterium sp*<br>*Pseudoalteromonas sp*<br>*Rahnella aquatilis*<br>*Vibrio logei*<br>*Vibrio tasmaniensis*<br>*Vibrio sp.*	*Hypocrea sp*<br>*Penicillium purpurogenum*	*Burkholderia glumae*<br>*Photobacter profundum*<br>*Vibrio logei*<br>*Vibrio sp.*	---
89	2007	---	---	NS	NS
92	2007	*Clostridium cadaveris*<br>*Clostridium perfringens*<br>*Cobetia sp.*<br>*Leeuwenhoekiella sp.*<br>*Vibrio sp.*	*Botrytis sp.*<br>*Epicoccum sp.*<br>*Cladosporium sp.*<br>*Penicillium sp.*	NS	NS
92	2008	---	---	*Clostridium perfringens*	*Alternaria sp.*<br>*Botrytis sp.*<br>*Epicoccum sp.*<br>*Cladosporium sp.*
92	2009	*Bacillus circulans*	---	---	---
101	2007	*Alteromonas sp.*<br>*Cobetia sp.*<br>*Marimonas sp.*<br>*Pseudomonas fluorescens*<br>*Vibrio sp.*	*Alternaria sp.*<br>*Botrytis sp.*<br>*Epicoccum sp.*<br>*Cladosporium sp.*<br>*Phialophora sp.*	NS	NS
101	2008	---	*Doratomyces sp.*	*Alteromonas sp.*<br>*Clostridium perfringens*<br>*Cobetia sp.*<br>*Halomonas sp.*<br>*Pseudoalteromonas sp.*	*Botrytis sp.*<br>*Cladosporium sp.*<br>*Phialophora sp.*
101	2009	*Bacillus sp.*<br>*Clostridium sordellii*<br>*Cobetia sp.*<br>*Halomonas sp.*<br>*Pseudoaltermonas sp.*<br>*Pseudomonas fluorescens*	*Cladosporium cladosporioides*<br>*Cladosporium sp.*	---	---
102	2007	*Clostridium cadaveris*<br>*Clostridium perfringens*<br>*Cobetia sp.*<br>*Marimonas sp.*<br>*Pseudomonas fluorescens*<br>*Vibrio sp.*	*Alternaria sp.*<br>*Botrytis sp.*<br>*Cladosporium sp.*<br>*Phialophora sp.*	NS	NS
102	2008	*marine proteobacterium*<br>*Pseudoalteromonas sp.*	*Fusarium sp.*	*Alteromonas sp.*<br>*Clostridium perfringens*<br>*Cobetia sp.*<br>*Halomonas sp.*<br>*Pseudoalteromonas sp.*	*Alternaria sp.*<br>*Aureobasidium sp.*<br>*Fusarium sp.*
102	2009	---	*Alternaria sp.*<br>*Epicoccum sp.*<br>*Cladosporium sp.*	---	---
103	2007	*Clostridium perfringens*<br>*Marimonas sp.*	*Alternaria sp.*<br>*Botrytis sp.*<br>*Epicoccum sp.*<br>*Cladosporium sp.*	NS	NS
103	2008	*Pseudoalteromonas sp.*	*Cladosporium sp.*	*Alteromonas sp.*<br>*Clostridium perfringens*<br>*Cobetia sp.*<br>*Halomonas sp.*<br>*Pseudoalteromonas sp.*<br>*Pseudomonas sp.*	*Botrytis sp.*<br>*Fusarium sp.*<br>*Cladosporium sp.*

103	2009	*Alteromonas sp.*<br>*Frigoribacterium sp.*<br>*Pseudomonas fluorescens*	*Alternaria sp.*<br>*Epicoccum sp.*<br>*Cladosporium sp.*	---	---
106	2007	NS	NS	*Clostridium perfringens*<br>*sponge bacterium*	*Alternaria sp.*<br>*Aureobasidium sp.*<br>*Botrytis sp.*<br>*Cladosporium sp.*<br>*Penicillium sp.*<br>*Phoma sp.*

Supplementary Table S2. Microbiota detected in control samples by year and source of sample.

2006	2007		2008	2009	
air	air	rain	human	air	rain
Alternaria sp.	*Aureobasidium pullulans*	*Alteromonas* sp.	*Arthrobacter* sp.	*Ascomycota* sp.	*Cryptococcus laurentii*
Hormodendrum cladosporioides	*Bacillus* sp.	*Exiguobacterium* sp.	*Paecilomyces variotii*	*Leewenhoekiella* sp.	*Hormodendrum* sp.
Penicillium glabrum	*Candida famata*	*Gelidibacter* sp.	*Staphylococcus epidermidis*	*Marinomonas blandensis*	*Penicillium* sp.
Penicillium namyslowski	marine proteobacterium	*Halomonas* sp.	*Staphylococcus lugdunesis*	*Micrococcus luteus*	*Rhodotorula mucilaginosa*
Trametes versicolour	*Shewanella hanedai*	*Moritella* sp.		*Penicillium* sp.	*Stentrophomonas maltophila*
	Staphylococcus capitis	*Pseudoalteromonas* sp.		*Pseudoalteromonas haloplanktis*	
		Rhodotorula mucilaginosa		*Pseudomonas guinea*	
		Vibrio sp.		*Psychromonoas arctica*	
		Vibrio spendidus		*Staphylococcus capitis*	
				Staphylococcus haemolyticus	

Supplementary Table S3. List of primers used in this study for PCR amplification.

Primer Name	Primer Sequence 5' - 3'	Species Specificity	Reference
vNS5	AAC TTA AAG GAA TTG ACG GAA G	Fungal 18S rDNA	[1]
NS6	GCA TCA CAG ACC TGT TAT TGC CTC	Fungal 18S rDNA	[1]
fD1	AGA GTT TGA TCC TGG CTC AG	Eubacteria 16S rDNA	[2]
rP2	ACG GCT ACC TTG TTA CGA CTT	Eubacteria 16S rDNA	[2]
Uni-C	AGA GTT TGA TCM TGG CTC AG	16S rRNA	[3]
Uni-D	GWA TTA CCG CGG CKG CTG	16S rRNA	[3]
FUN-A	TGG AGG GCA AGT CTG GTG	Fungal 18S	this study
FUN-B	AAC TAA GAA CGG CCA TGC AC	Fungal 18S	this study
FUN-1	GTA TAT TAA AGT TGT TGC AG	Fungal 18S	this study
FUN-2	TGT CTG GAC CTG GTG AG	Fungal 18S	this study
BY-1	GAA GAT GCT GAC AGT CTC GTG	Canine Distemper	this study
BY-4	CAA CTA TCC CCA TTC CAT GTG	Canine Distemper	this study
BY-2	TCT GGC GAA GAT TAT TCC G	Canine Distemper	this study
BY-3	TCC CTA CAT TTC TGC TTG TCC	Canine Distemper	[4]
C-1	TGG CTC GGT TGC CAA TAT CAA	*Brucella* spp.	[5]
C-2	CGC GCT TGC CTT TCA GGT CTG	*Brucella* spp.	[5]
DOD-1	ATT AAA AAG GGS ACA GGA GAG AGA TCA GCC	Morbillivirus	[6]
DOD-2	ATT GGG TTG CAC CAC TTG TC	Morbillivirus	[6]
GPO-1	ACT CCT ACG GGA GGC AGC AGT A	Mollicutes	[7,8]
GPO-2	TGC ACC ATC TGT CAC TCT GTT AAC CTC	Mollicutes	[7,8]
GPO-3	GGG AGC AAA CAG GAT TAG ATA CCC	Mollicutes	[7,8]
GPO-4	CTT AAA GGA ATT GAC GGG AAC CCG	Mollicutes	[7,8]
M +25	AGA TGA GTC TTC TAA CCG AGG TCG	Influenza A	[4]
M -124	TGC AAA AAC ATC TTC AAG TCT CTG	Influenza A	[4]
M +64	FAM-TCA GGC CCC CTC AAA GCC GA-TAMRA	Influenza A	[4]

Supplementary Table S4. Pair-wise comparisons between sample sources (SML, breath, controls) by analysis of similarity (ANOSIM) and permutational analysis of variance (PERMANOVA) results of detected microbes.

**Years**	**Comparison**	**ANOSIM R**	**ANOSIM significance (%)**	**PERMANOVA t**	**p (perm)**
**All years**	Breath *vs* SML	0.202	0.1	2.5952	0.001
	Breath *vs* controls	0.057	18.8	1.0865	0.275
	SML *vs* controls	0.221	0.2	1.9314	0.001
**2006**	Breath *vs* SML	0.015	37.4	1.3224	0.098
	Breath *vs* controls	0.135	27.5	1.1702	0.181
	SML *vs* controls	0.007	43.0	1.174	0.207
**2007**	Breath *vs* SML	0.781	0.1	2.9261	0.001
	Breath *vs* controls	-0.069	69.1	1.1698	0.166
	SML *vs* controls	0.918	0.1	2.9774	0.002
**2008**	Breath *vs* SML	0.906	0.2	2.8642	0.003
	Breath *vs* controls	0.000	46.7	1.338	0.218
	SML *vs* controls	0.892	1.1	2.4308	0.009
**2009**	Breath *vs* SML	0.226	2.5	2.1696	0.002
	Breath *vs* controls	0.46	2.4	1.252	0.074
	SML *vs* controls	0.384	0.3	1.7518	0.017

Supplementary Table S5. Pair-wise analysis of similarity (ANOSIM) R values for breath and control samples by year. R values range from 1 (no similarity between groups) to -1 (complete similarity between groups). Values in bold have a significance value < 0.05).

	2006, breath	2006, control	2007, breath	2007, control	2008, breath	2008, control	2009, breath	2009, control
2006, breath								
2006, control	0.135							
2007, breath	-0.090	-0.122						
2007, control	0.098	0.231	-0.069					
2008, breath	**0.520**	**0.843**	0.197	**0.613**				
2008, control	0.370	0.583	0.070	0.491	0.000			
2009, breath	**0.367**	**0.827**	**0.244**	**0.621**	**1.000**	**1.000**		
2009, control	**0.534**	0.944	**0.621**	0.333	**1.000**	1.000	**0.46**	

Supplementary Table S6. Bray-Curtis matrix for exhaled breath, sea surface microlayer (SML), and corresponding control samples used in microbial community analyses.

Supplementary Figure S1. Taxonomics of identified microbes in SRKW breath and SML.

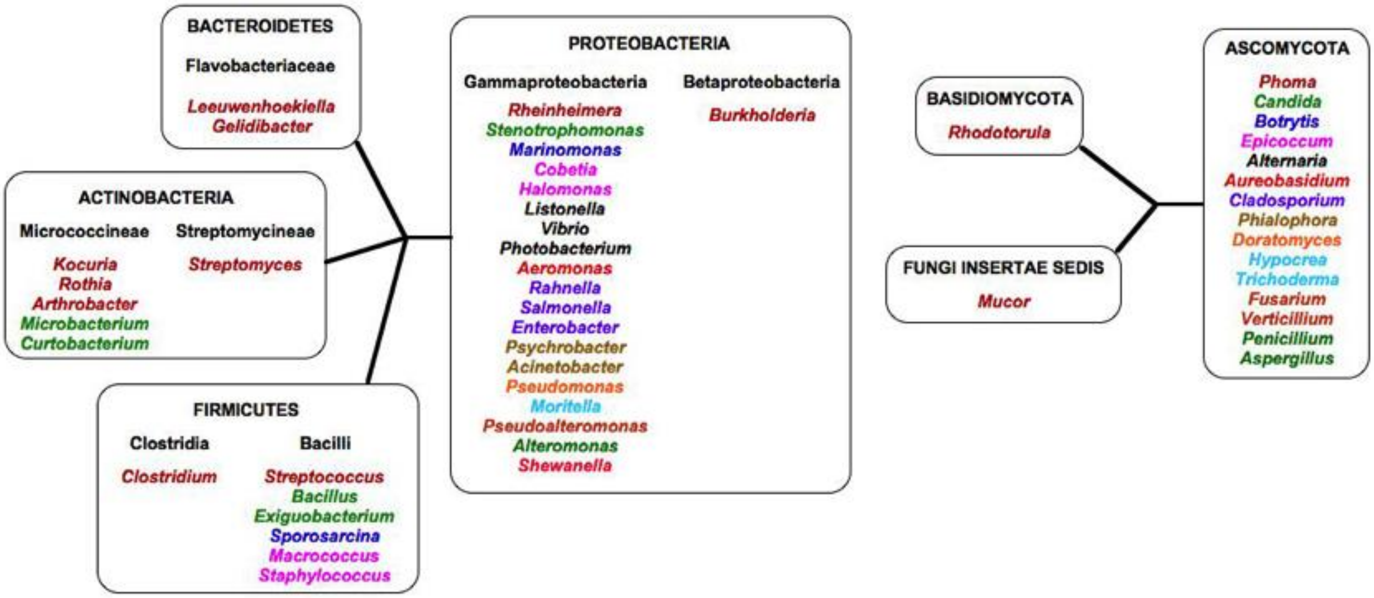

Supplementary Figure S2. Sampling exhaled breath from SRKW. (A) Petri dishes mounted on extensible pole, showing lids in place to maintain sterility. (B) Position of pole with petri dishes to collect exhaled whale breath.

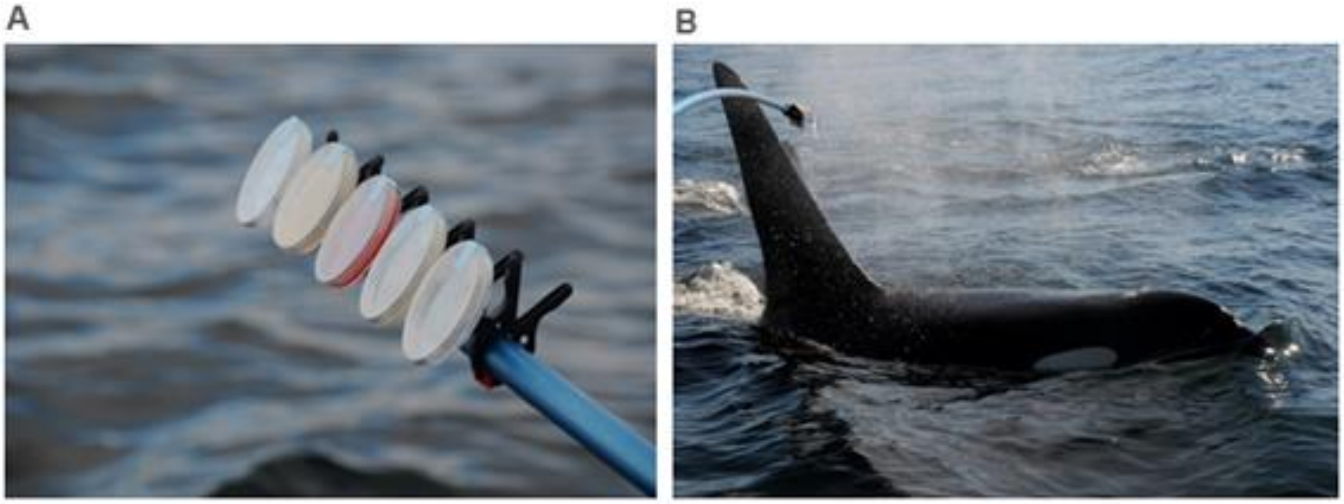

Supplementary Figure S3. Demonstration of SML sampling, showing scientist using a silicone squeegee (left hand) to direct water into plastic funnel from glass plate mounted on suction cup grip (right hand). Glass plate is repeatedly placed on surface to collect SML until target volume is obtained.

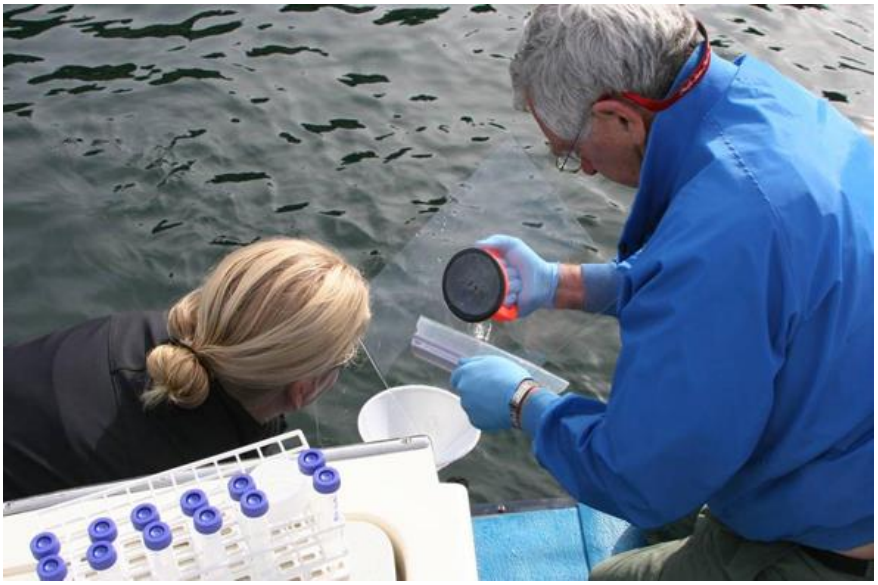

References

1 Wu, Z., Wang, X. R. & Blomquist, G. Evaluation of PCR primers and PCR conditions for specific detection of common airborne fungi. *J Environ Monit* **4**, 377-382 (2002).

2 Weisburg, W. G., Barns, S. M., Pelletier, D. A. & Lane, D. J. 16S ribosomal DNA amplification for phylogenetic study. *J Bacteriol* **173**, 697-703 (1991).

3 Lane, D. J. in *Nucleic Acid Techniques in Bacterial Systematics* (eds E. Stackebrandt & M. Goodfellow) 115-175 (Wiley, 1991).

4 Spackman, E. *et al.* Development of a real-time reverse transcriptase PCR assay for type A influenza virus and the avian H5 and H7 hemagglutinin subtypes. *J Clin Microbiol* **40**, 3256-3260 (2002).

5 Casanas, M. C. *et al.* Specificity of a polymerase chain reaction assay of a target sequence on the 31-kilodalton *Brucella* antigen DNA used to diagnose human brucellosis. *Eur J Clin Microbiol Infect Dis* **20**, 127-131 (2001).

6 Krafft, A. *et al.* Postmortem diagnosis of morbillivirus infection in bottlenose dolphins (*Tursiops truncatus*) in the Atlantic and Gulf of Mexico epizootics by polymerase chain reaction-based assay. *J Wildl Dis* **31**, 410-415, doi:10.7589/0090-3558-31.3.410 (1995).

7 van Kuppeveld, F. J. *et al.* Genus- and species-specific identification of mycoplasmas by 16S rRNA amplification. *Appl Environ Microbiol* **58**, 2606-2615 (1992).

8 van Kuppeveld, F. J. *et al.* Detection of *Mycoplasma* contamination in cell cultures by a mycoplasma group-specific PCR. *Appl Environ Microbiol* **60**, 149-152 (1994).
